# Chromosomal phylogeny and comparative chromosome painting among *Neacomys* species (Rodentia, Sigmodontinae) from eastern Amazonia

**DOI:** 10.1186/s12862-019-1515-z

**Published:** 2019-10-10

**Authors:** Willam Oliveira da Silva, Julio Cesar Pieczarka, Marlyson Jeremias Rodrigues da Costa, Malcolm Andrew Ferguson-Smith, Patricia Caroline Mary O’Brien, Ana Cristina Mendes-Oliveira, Rogério Vieira Rossi, Cleusa Yoshiko Nagamachi

**Affiliations:** 1 Centro de Estudos Avançados da Biodiversidade, Laboratório de Citogenética, Instituto de Ciências Biológicas, Universidade Federal do Pará (UFPA), Belém, Pará Brazil; 2 Cambridge Resource Centre for Comparative Genomics, Department of Veterinary Medicine, University of Cambridge, Cambridge, UK; 3 Laboratório de Zoologia e Ecologia de Vertebrados, ICB, Universidade Federal do Pará (UFPA), Belém, Brazil; 4 Instituto de Biociências, Universidade Federal do Mato Grosso (UFMT), Cuiabá, Mato Grosso Brazil

**Keywords:** Karyotypic diversity, ZOO-FISH, Akodontini, Oryzomyini, Amazon biodiversity

## Abstract

**Background:**

The *Neacomys* genus is predominantly found in the Amazon region, and belongs to the most diverse tribe of the Sigmodontinae subfamily (Rodentia, Cricetidae, Oryzomyini). The systematics of this genus and questions about its diversity and range have been investigated by morphological, molecular (Cytb and COI sequences) and karyotype analysis (classic cytogenetics and chromosome painting), which have revealed candidate species and new distribution areas. Here we analyzed four species of *Neacomys* by chromosome painting with *Hylaeamys megacephalus* (HME) whole-chromosome probes, and compared the results with two previously studied *Neacomys* species and with other taxa from Oryzomyini and Akodontini tribes that have been hybridized with HME probes. Maximum Parsimony (MP) analyses were performed with the PAUP and T.N.T. software packages, using a non-additive (unordered) multi-state character matrix, based on chromosomal morphology, number and syntenic blocks. We also compared the chromosomal phylogeny obtained in this study with molecular topologies (Cytb and COI) that included eastern Amazonian species of *Neacomys*, to define the phylogenetic relationships of these taxa.

**Results:**

The comparative chromosome painting analysis of the seven karyotypes of the six species of *Neacomys* shows that their diversity is due to 17 fusion/fission events and one translocation, pericentric inversions in four syntenic blocks, and constitutive heterochromatin (CH) amplification/deletion of six syntenic autosomal blocks plus the X chromosome. The chromosomal phylogeny is consistent with the molecular relationships of species of *Neacomys*. We describe new karyotypes and expand the distribution area for species from eastern Amazonia and detect complex rearrangements by chromosome painting among the karyotypes.

**Conclusions:**

Our phylogeny reflects the molecular relationships of the Akodontini and Oryzomyini taxa and supports the monophyly of *Neacomys*. This work presents new insights about the chromosomal evolution of this group, and we conclude that the karyotypic divergence is in accord with phylogenetic relationships.

## Background

The genus *Neacomys* Thomas, 1900 comprises 12 recognized species distributed from Panama to central Brazil and Bolivia; these rodents are mainly found in the Amazon region, and belong to the most diverse tribe of the Sigmodontinae subfamily (Rodentia, Cricetidae, Oryzomyini) [1–9]. Recently, morphologic, molecular and cytogenetic analyses have revealed candidate species along with, new distributions and karyotypes; this work has reshaped the geographic boundaries, raised questions regarding the taxonomic status, suggested the occurrence of species complexes in some lineages (e.g., *N. minutus*) and reinforced the view that this genus is more diverse than was previously believed [2, 5–10].

Cytogenetic studies on *Neacomys* show a range of diploid number (2n) from 28 to 64, and an autosomal fundamental number (FN) from 36 to 70 (Table 1), but in most species the diploid and fundamental number are the only available information, and the results of other cytogenetic techniques are not reported. Recent studies using classic banding, FISH with telomeric and rDNA 18S probes define five new karyotypes [5], two for *N. paracou* (2n = 56, FN = 62; 66), one for *N. dubosti* (2n = 64/FN = 68) and two for an undescribed species, *Neacomys* sp. (2n = 58/FN = 64; 70). A third karyotype was described for this *Neacomys* sp., which has the same 2n (58), but a different FN (66) [8].

**Table 1:** Localities/distribution of species of *Neacomys* with chromosomal data available in literature and karyotyped specimens in the present study. (2n) diploid number, (FN) autosomal fundamental number. Numbers in parentheses refer to localities shown in Fig. 1. Brazilian (BR) states are Amapá (AP), Mato Grosso (MT), and Pará (PA). Species analyzed with chromosome painting in the present study are bolded in the leftmost column

Species	Karyotype	Distribution/Localities	Reference
***Neacomys*** **sp. C**	2n = 58, FN = 64	(1) Marabá (BR, PA)	Da Silva et al. [5]
*Neacomys* sp. C^a^	2n = 58, FN = 66	Vila Rica (BR, MT)	Di-Nizo et al. [8]
***Neacomys*** **sp. D**	2n = 58, FN = 70	(2) Afuá (Marajó island) and (3) Chaves (Marajó island), BR. PA	Present study, Da Silva et al. [5]
***N. paracou***	2n = 56, FN = 64	(4) Mazagão (BR, AP)	Present study
*N. paracou*	2n = 56, FN = 62, 66	Southeast of Venezuela, Suriname, French Guiana and Brazil (South of Roraima, northeast of Amazonas, northwest of Pará and Amapá states)	Voss et al. [3]; Da Silva et al. [5]
***Neacomys*** **sp. E**	2n = 62, FN = 60	(5) Santa Bárbara (BR, PA)	Present study
*N. dubosti*	2n = 62/64, FN = 68	Southeast of Suriname, French Guiana and Brazil (Amapá state)	Voss et al. [3]; Da Silva et al. [5]
***N. amoenus***	2n = 64, FN = 68	(6) Alta Floresta (BR, MT)	Present study
*N. amoenus* ^b^	2n = 64, FN = 68	In the Cerrado, between eastern Bolivia and western Brazil, and in Amazon forest, from southeast of Ecuador to northeast of Peru	Patton et al. [2]; Hurtado and Pacheco [6]
*Neacomys* sp. A	2n = 58, FN = 68	(7, 8) Itaituba (BR, PA) and Jacareacanga (BR, PA)	Oliveira da Silva et al. [7]
*Neacomys* sp. B	2n = 54, FN = 66	(9) Juruti (BR, PA) and (10) Itaituba (BR, PA)	Oliveira da Silva et al. [7]
*N. guianae*	2n = 56	Guianas, south of Venezuela and Brazil (north of Roraima state)	Baker et al. [1]; Voss et al. [3]
*N. minutus*	2n = 35–36, FN = 40	Brazil (southwest of Amazonas state, only in lower and middle Juruá River)	Patton et al. [2]
*N. musseri*	2n = 34, FN = 64–68	Southeast of Peru and western Brazil (northwest of Acre state, at Rio Juruá River source)	Patton et al. [2]
*N. tenuipes*	2n = 56	North of Venezuela, and west of Colombia	Redi et al. [4]
*N. rosalindae*	2n = 48/FN = 50	Northeastern of Peru and eastern of Ecuador	Sánchez-Vendizú et al. [9]
*N. macedoruizi*	2n = 28/FN = 36	Tingo María National Park, Peru	Sánchez-Vendizú et al. [9]

^a^Referred as *Neacomys* sp. by Di-Nizo et al. [8]; ^b^Referred as *N. spinosus* by Patton et al. [2]

Two additional undescribed species identified by karyotypic and molecular data (Cytb and COI sequences): *Neacomys* sp. A (2n = 58/FN = 68) and *Neacomys* sp. B (2n = 54/FN = 66) [7], were analyzed using whole-chromosome probes from *Hylaeamys megacephalus* (HME) [11].

The authors linked the findings in these two species with data on three other species mentioned above [5], and defined four synapomorphies for the genus (chromosomal associations HME 6a/21, (9,10)/7b/(9,10), 12/(16,17), and 20/(13,22)/4), that agreed with the chromosomal evolutionary pattern previously described for eastern Amazonian species [5], with fusion/fission, pericentric inversions and amplification/deletion of constitutive heterochromatin events as the main cause of the karyotypic diversity.

The *Neacomys* genus was always recovered as a monophyletic group by the use of different markers (morphological and/or molecular - Cytb, COI, IRBP) and approaches (Maximum Parsimony, Maximum Likelihood and Bayesian Inference), with values above 74% of support and variation according to the analysis and set of data, but most of the investigations were performed in western Brazilian Amazonian samples [2, 5–7, 9, 12–14].

A molecular analysis of nine species from eastern Amazonia was performed [7], including the three undescribed taxa mentioned above: *Neacomys* sp. [5], *Neacomys* sp. A and *Neacomys* sp. B [7]. The resulting phylogeny demonstrated that, despite the new species, the main relationships among the species remain the same: *N. paracou* stands as a sister to the other species of the genus, followed by *N. spinosus* and two clades – one comprising *N. dubosti* and species from Eastern Amazonia (*Neacomys* sp. A, *Neacomys* sp. B and *Neacomys* sp.) and the other formed by *N. minutus*, *N. musseri* and *N. guianae*.

In an effort to understand the direction of chromosome change, Da Silva et al. [5] plotted the 2n and FN data on a molecular phylogeny (Cytb) and discussed the chromosomal evolution of the genus. The authors observed that the clades were structured with distinct chromosomal evolutionary patterns, and postulated that three major and independent events had occurred: two increases and one reduction from the hypothetical ancestral 2n (56). They also proposed that Robertsonian translocations were the main cause of the 2n reductions observed in *N. minutus* (2n = 35–36/FN = 40) and *N. musseri* (2n = 34/FN = 64–68) [2], but the differences in FN were caused by centromeric repositioning. The same events probably occurred during the karyotypic evolution of *N. rosalindae* (2n = 48/FN = 50) and *N. macedoruizi* (2n = 28/FN = 36) [9]. The independent occurrence of both increases and decreases of 2n during the chromosomal evolution of *Neacomys* (see [5]) is unusual, since a reduction of 2n is the evolutionary trend seen in other taxa (e.g., *Akodon*) [15].

Comparative chromosome painting for the reconstruction of phylogenies is another approach that has contributed to the knowledge on systematics and karyotypic evolution of several groups of mammals [16], including bats [17–20] and rodents from the *Akodon* genus [21].

**Fig. 1:**
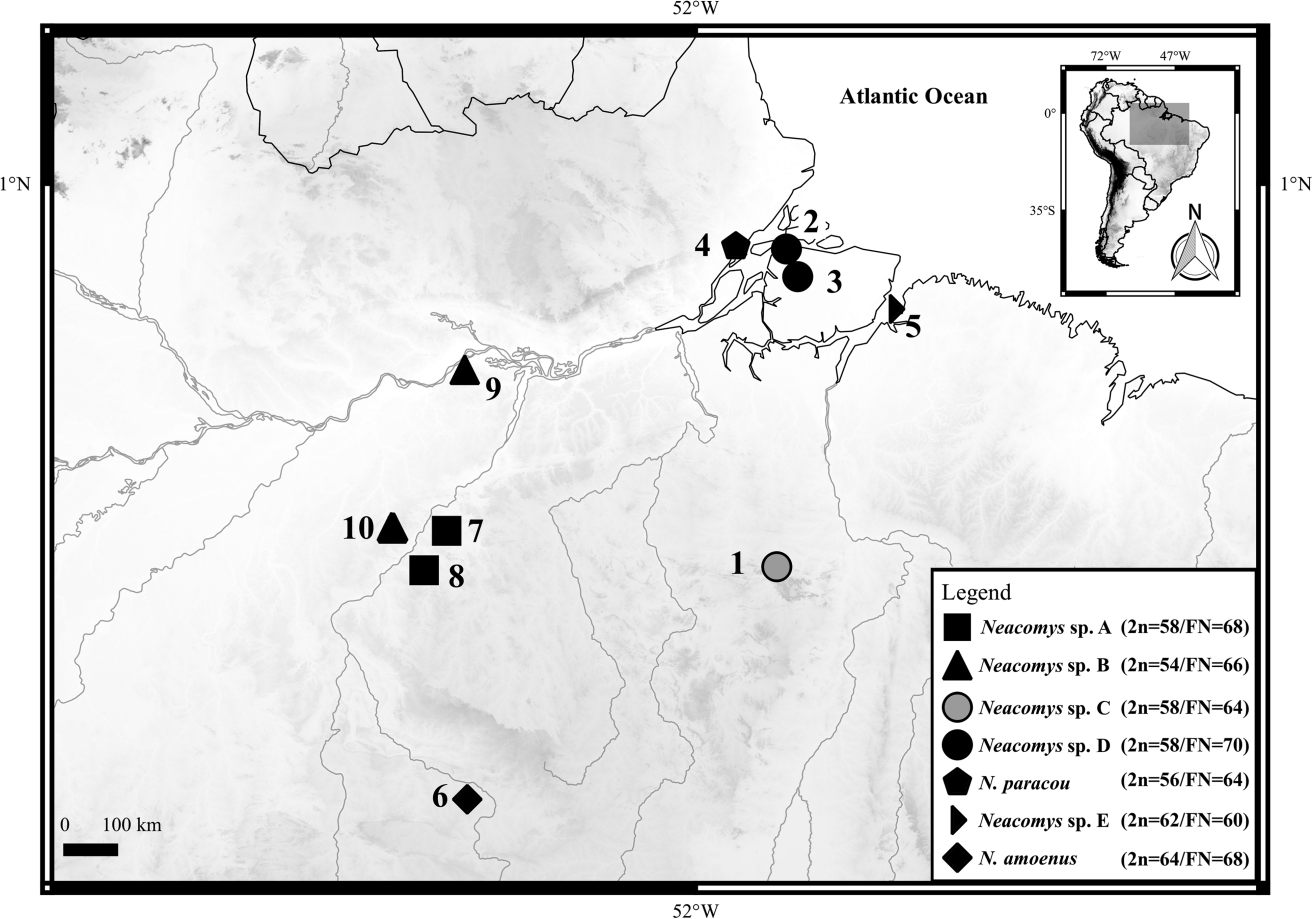
Map showing collection points of samples of *Neacomys*. Diploid number (2n) and fundamental number (FN) are shown based on Table 1. The numbers refer to localities mentioned in Table 1. (1) Marabá – PA; (2) Afuá, Marajó Island – PA; (3) Chaves, Marajó Island – PA; (4) Mazagão – AP; (5) Santa Bárbara – PA; (6) Alta Floresta – MT; (7) Itaituba – PA; (8) Jacareacanga – PA; (9) Juruti – PA; (10) Itaituba – PA. Brazilian states are Amapá (AP), Mato Grosso (MT) and Pará (PA). This map was made using QUANTUM-GIS (QGIS) program version 2.10.1. Database was obtained from DIVA and REDLIST

Given the high chromosomal variation, the presence of distinct evolutionary pathways and the phylogenetic patterns found in *Neacomys*, the aim of this study is to use chromosome painting with HME probes [11] to examine the chromosomal events leading to the karyotypic diversity of five eastern Amazonian taxa of *Neacomys*: *Neacomys* sp. C, *Neacomys* sp. D, *Neacomys* sp. E, *N. paracou*, and *N. amoenus* (Fig. 1); comparing them with other taxa previously hybridized with HME probes [7, 11, 22–24], and investigate if the karyotypic divergence followed the phylogenetic relationships. This will allow the reconstruction of chromosomal homologies for comparison with the molecular phylogenies that include eastern Amazonian species of *Neacomys* [7].

## Results

### Classic and molecular cytogenetics

**Fig. 2:**
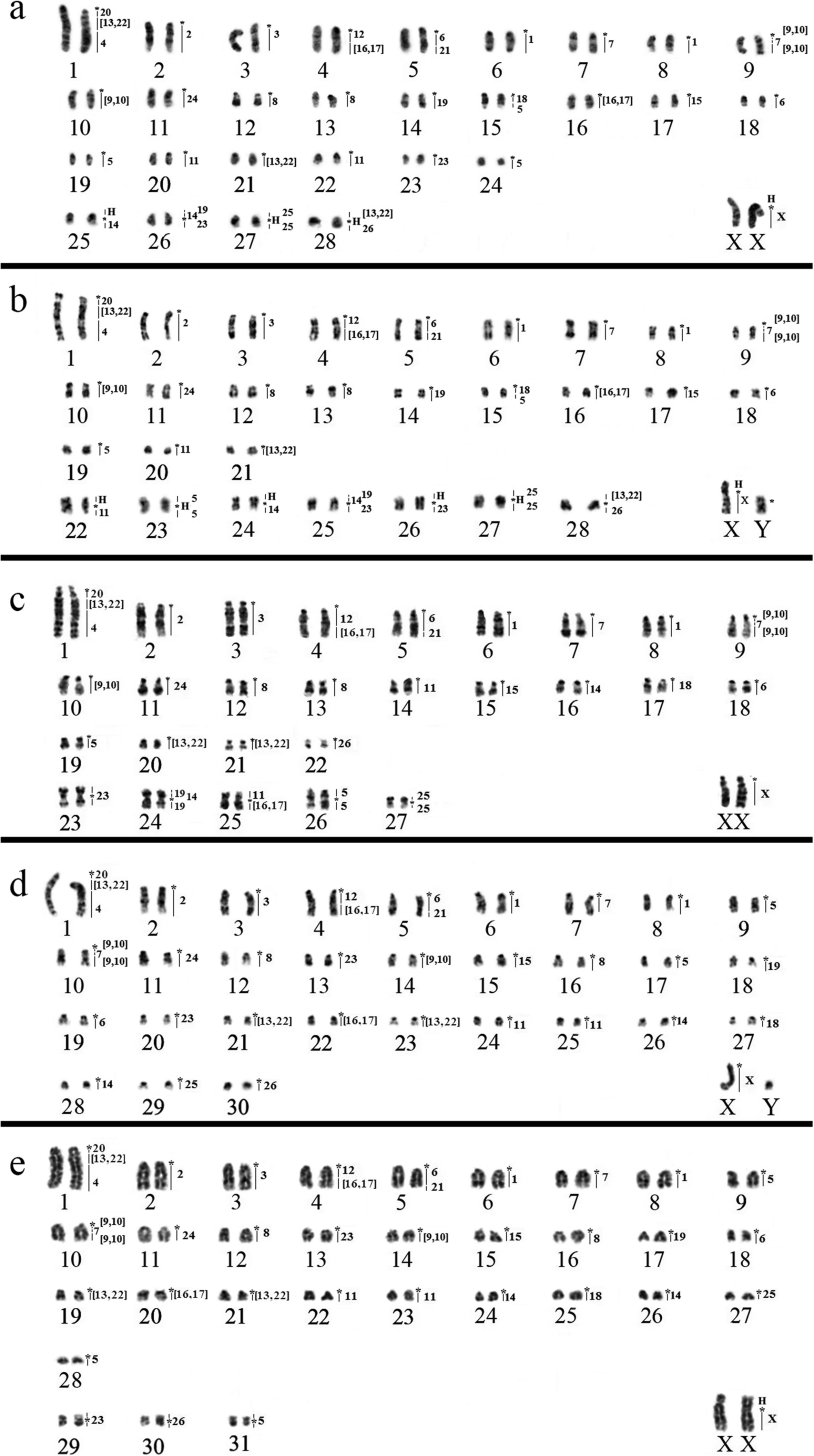
G-banding with homologies revealed by *Hylaeamys megacephalus* (HME) probes [11]. **a** *Neacomys* sp. C (NSP-C, 2n = 58/FN = 64); **b** *Neacomys* sp. D (NSP-D, 2n = 58/FN = 70); **c** *N. paracou* (NPA, 2n = 56/FN = 64); **d** *Neacomys* sp. E (NSP-E, 2n = 62/FN = 60); **e** *N. amoenus* (NAM, 2n = 64/FN = 68). (*) Indicates centromere. (H) Indicates large block of constitutive heterochromatin

**Fig. 3:**
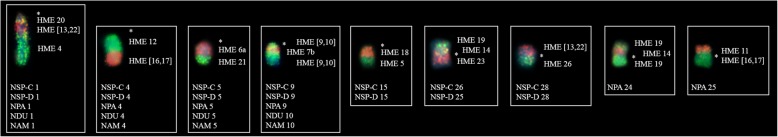
Chromosomal associations in species of *Neacomys*. Numbers on right correspond to homologies revealed by *Hylaeamys megacephalus* (HME) probes [11]. Abbreviations and numbers below correspond to the chromosomal pair from each species that show the respective chromosomal association. (*) Indicates centromere. Legend: *Neacomys* sp. C (NSP-C), *Neacomys* sp. D (NSP-D), *N. paracou* (NPA), *Neacomys* sp. E (NSP-E), and *N. amoenus* (NAM)

The chromosome painting analysis performed with HME probes on species of *Neacomys* showed that centromeric (*) and heterochromatic regions do not present hybridization signals. The results are detailed in Table 2 and Figs. 2 and 3.

**Table 2:** Chromosomal homology among species hybridized with HME whole chromosome probes

HME	CLA	TNI	AMO	ASP	NLA	OCA-PA	OCA-RJ	NSP-A	NSP-B	NSP-C	NSP-D	NPA	NSP-E	NAM
1	2q, 20	4, 8	1q dist., 4q	1q int., 2q dist.	5q dist., 7	13, 16, 29	13, 17, 28	6, 8	2p, 4q	6, 8	6, 8	6, 8	6, 8	6, 8
2	10, 18, 19	7, 12	1q int., 7q	1p dist., 2q int.	9q int., 13	4	1p, 4	2	3q	2	2	2	2	2
3	1q int., 3p	1 int. and dist.	2q	2p dist.	3q dist.	1	1q	3	2p	3	3	3	3	3
4	5, 13	13, 15	1p prox., 5p dist.	2q prox. and int., 3q int.	1q prox., 10q prox., 11q dist.	2	2	1q dist.	1q dist.	1q dist.	1q dist.	1q dist.	1q dist.	1q dist.
5	1p dist., 1q prox., 8	2 dist., 5 prox., 6 prox.	3q int., 6p int., 10	1p int. and prox., 3q int. (ts)	6q dist., 12q int., 14q dist.	3q dist., 15, 27	3q dist., 15, 27	19, 22, 24, 26p	3p dist., 21, 22, 24	15q dist., 19, 24	15q dist., 19, 23	19, 26	9, 17	9, 28, 31
6	4q dist.	3 prox. and int.	2p	2p int.	2q int.	9, 25, 26	9, 16	5q prox., 18	5q prox., 8	5q prox., 18	5q prox., 18	5q prox., 18	5q prox., 19	5q prox., 18
7	3q int.	18	5q prox., 8q	1p int., 3q int.	1q int., 4q prox.	7	7	7, 9q int.	3p int., 5p	7, 9q int.	7, 9q int.	7, 9q int.	7, 10q int.	7, 10q int.
8	4q prox., 7	6 dist.	3p	3q prox.	1q int.	5	5	12, 13	6, 15	12, 13	12, 13	12, 13	12, 16	12, 16
(9,10)	2p dist., 3q dist.	2 prox., 5 dist.	5q, 9p	1q int., 3q int.	1q dist., 6q prox.	3q prox., 12	3q prox., 12	9q (ts), 10	1p, 3p int. (ts)	9q (ts), 10	9q (ts), 10	9q (ts), 10	10q (ts), 14	10q (ts), 14
11	11q prox., 6	9 dist., 10 prox.	1p int., 6q dist.	2q int., 3q dist.	10q int., 12q dist.	6q dist., 8 dist.	6q dist.; 8q dist.	20, 23p	11, 26	20, 22	20, 22q	14, 25p	24, 25	22, 23
12	2p prox.	16	1q int.	2q int.	5q prox.	14	14	4q prox.	4q prox.	4q prox.	4q prox.	4q prox.	4q prox.	4q prox.
(13,22)	1q (ts), 9	9 prox., 11 int. and dist., 21	3q prox., 4p dist., 6q prox.	1q int., 3q int. (ts)	4q dist., 12q prox., 14q prox.	10q dist., 18q prox., 22	10q dist., 19q prox., 22	1q int., 26q, 27p	1q int., 19, 23	1q int., 21, 28p	1q int., 21, 28p	1q int., 20, 21	1q int., 21, 23	1q int., 19, 21
14	1p int., 21	17 prox., 24	6p prox., 8p int.	1p int., 3q int.	2q int., 15q int.	3q int., 30	3q int., 29	23q, 25p prox.	20, 25	25q, 26p prox.	24q, 25p prox.	16, 24p prox.	26, 28	24, 26
15	12	19	9q	1q dist.	8q dist.	11	11	15	9	17	17	15	15	15
(16,17)	1q prox., 11q dist.	10 dist., 22	1p dist., 3q dist.	2p prox., 2q int., 3q int.	2q prox., 10q dist.	19, 20	20, 24	4q dist., 16	4q dist., 10	4q dist., 16	4q dist., 16	4q dist., 25q	4q dist., 22	4q dist., 20
18	16	1 prox., 23	1q prox., 5p prox.	2p int., 2q int., 3q int.	3q prox., 9q prox.	21	21	17	7	15q prox.,	15q prox.	17	27	25
19	1p int., 3q prox.	17 dist.	8p dist., 8q prox.	1p int. (ts)	15q prox. and dist.	6q int., 28	6q int., 26	14, 25p dist.	18	14, 26p dist.	14, 25p dist.	24p dist., 24q	18	17
20	1q dist.	11 prox.	4q prox.	1q prox. and int.	4q int.	10q prox.	10q prox.	1q prox.	1q prox.	1q prox.	1q prox.	1q prox.	1q prox.	1q prox.
21	4p, 4q int.	3 dist.	2p dist.	2p int.	2q dist.	18q dist.	19q dist.	5q dist.	5q dist.	5q dist.	5q dist.	5q dist.	5q dist.	5q dist.
23	15	20	7p	1p int.	8q prox.	6q prox., 23	6q prox., 23	21, 25q	14, 16	23, 26q	25q, 26q	23	13, 20	13, 29
24	14	14	6p dist.	3q int. (ts)	9q dist.	17	18	11	12	11	11	11	11	11
25	17	1 prox.	2p prox.	2p int., 3q int.	3q int., 11q prox.	24	25	28	13	27	27	27	29	27
26	22	25	11	4	16	8q prox.	8q prox.	27q	17	28q	28q	22	30	30
X	X	X	X (Xq)	X	X	Xq	Xq	Xq	X	Xq	Xq	X	Xq	Xq
Total	40 signs	36 signs	38 signs	45 signs	40 signs	38 signs	38 signs	40 signs	39 signs	39 signs	39 signs	37 signs	37 signs	38 signs

*p* Short arm, *q* Long arm, *prox* Proximal, *int* Interstitial, *dist* Distal, *ts* Two segments, *HME Hylaeamys megacephalus*, *CLA Cerradomys langguthi* [11], *TNI Thaptomys nigrita*, *AMO Akodon montensis* [22], *ASP Akodon* sp., *NLA Necromys lasiurus* [23], *OCA-PA O. catherinae-*Pará, *OCA-RJ O. catherinae-*Rio de Janeiro [24], *NSP-A Neacomys* sp. A, *NSP-B Neacomys* sp. B [7], *NSP-C Neacomys* sp. C, *NSP-D Neacomys* sp. D, *NPA N. paracou*, *NSP-E Neacomys* sp. E, and *NAM N. amoenus*

### *Neacomys* sp. C (NSP-C, 2n = 58/FN = 64) and *Neacomys* sp. D (NSP-D, 2n = 58/FN = 70)

*Neacomys* sp. C karyotype (NSP-C, 2n = 58/FN = 64, Fig. 2a) was previously published with classic banding [5], for specimens collected in Marabá (PA) identified as “*Neacomys* sp.”. Here we performed chromosome painting with HME probes in this karyotype.

The *Neacomys* sp. D karyotype (NSP-D, 2n = 58/FN = 70, Fig. 2b) was previously published with classic banding [5], for specimens collected in Chaves town, Marajo Island (PA). Here, we performed chromosome painting with HME probes in this karyotype. We collected an additional sample from Afuá, Marajó Island, which has constitutive heterochromatin (CH) at the centromeric region of almost all autosomes, with large CH blocks in five meta/submetacentric pairs and the X chromosome. The Y chromosome is almost entirely heterochromatic (Additional file 1: Figure S1a). So, the sample of *Neacomys* sp. D from Afuá shows the same karyotype of *Neacomys* sp. D from Chaves, and they presented no distinction on the chromosome painting analysis. The divergence between the FN of NSP-C and NSP-D is due to the presence of large CH blocks in the short arms of NSP-D 22, 23, and 26 (metacentrics, Additional file 1: Figure S1a), whereas these blocks are absent in NSP-C 22, 24, and 23 (acrocentrics), respectively.

Twelve of the 24 chromosomal probes show conserved synteny (11 autosomal probes plus the X chromosome), in which five (HME 2, 3, 15, 24 and 25) hybridize to whole chromosomes of NSP-C (2, 3, 17, 11 and 27, respectively) and NSP-D (2, 3, 17, 11 and 27, respectively) and six (HME 4, 12, 18, 20, 21 and 26) hybridize to parts of other chromosomes in NSP-C (1q distal, 4q proximal, 15q proximal, 1q proximal, 5q distal and 28q, respectively) and NSP-D (1q distal, 4q proximal, 15q proximal, 1q proximal, 5q distal and 28q, respectively). The other 12 autosomal probes show more than one signal in NSP-C and NSP-D, with ten (HME 1, 6, 7, 8, (9,10), 11, 14, (16,17), 19 and 23) hybridizing to two chromosomes each; HME 5 and (13,22) show signals in three chromosomes each. In NSP-D the short arms of pairs 22, 23, and 26 did not hybridize to any HME probes because they are heterochromatic (Additional file 1: Figure S1a). Seven NSP-C and NSP-D pairs show association of syntenic blocks to multiple HME probes (Fig. 3, Table 2).

### *Neacomys paracou* (NPA, 2n = 56/FN = 64)

*Neacomys paracou* karyotype has 2n = 56/FN = 64 with autosomes comprising 22 acrocentric pairs and five meta/submetacentric pairs; the X chromosome is a middle-sized acrocentric and the Y chromosome is a small-sized acrocentric (Fig. 2c; Additional file 1: Figure S1b). The CH is distributed at the centromeric region of all autosomes and the X chromosome. The Y chromosome is almost entirely heterochromatic (Additional file 1: Figure S1b).

Fourteen of the 24 chromosomal probes show conserved synteny (13 autosomal probes plus the X chromosome), in which eight (HME 2, 3, 15, 18, 23, 24, 25 and 26) hybridize to whole chromosomes of NPA (2, 3, 15, 17, 23, 11, 27 and 22, respectively) and five (HME 4, 12, 19, 20 and 21) hybridize to parts of other chromosomes (NPA 1q distal, 4q proximal, 24p distal e 24q, 1q proximal and 5q distal, respectively). The other ten autosomal probes show more than one signal in NPA, with nine (HME 1, 5, 6, 7, 8, (9,10), 11, 14 and (16,17)) hybridizing to two chromosomes each; HME (13,22) gives signals in three chromosomes. Six NPA pairs show association of syntenic blocks to multiple HME probes (Fig. 3, Table 2).

### *Neacomys* sp. E (NSP-E, 2n = 62/FN = 60)

*Neacomys* sp. E has 2n = 62/FN = 60 with autosomes comprising 30 acrocentric pairs; the X chromosome is a middle-sized acrocentric and the Y chromosome is a small-sized acrocentric (Fig. 2d). The CH is distributed at the centromeric region of all autosomes and the X chromosome. The Y chromosome is almost entirely heterochromatic (Additional file 1: Figure S1c).

Thirteen of the 24 chromosomal probes show conserved synteny (12 autosomal probes plus the X chromosome), in which eight (HME 2, 3, 15, 18, 19, 24, 25 and 26) hybridize to whole chromosomes of NSP-E (2, 3, 15, 27, 18, 11, 29 and 30, respectively) and four (HME 4, 12, 20 and 21) hybridize to parts of other chromosomes (NSP-E 1q distal, 4q proximal, 1q proximal and 5q distal, respectively). The other 11 autosomal probes give more than one signal in NSP-E, with ten (HME 1, 5, 6, 7, 8, (9,10), 11, 14, (16,17) and 23) hybridizing to two chromosomes each; HME (13,22) shows signals in three chromosomes. Four NSP-E pairs show association of syntenic blocks to multiple HME probes (Fig. 3, Table 2).

### *Neacomys amoenus* (NAM, 2n = 64/FN = 68)

The karyotype of *Neacomys amoenus* has 2n = 64/FN = 68 with autosomes comprising 28 acrocentric pairs and three small metacentric pairs; the X chromosome is a middle-sized submetacentric and the Y chromosome is a small-sized acrocentric (Fig. 2e). CH is distributed at the centromeric region of all autosomes. The X chromosome has a large CH block in the short arm and the Y chromosome is almost entirely heterochromatic (Additional file 1: Figure S1d).

Thirteen of the 24 chromosomal probes show conserved synteny (12 autosomal probes plus the X chromosome), in which eight (HME 2, 3, 15, 18, 19, 24, 25 and 26) hybridize to whole chromosomes of NAM (2, 3, 15, 25, 17, 11, 27 and 30, respectively) and four (HME 4, 12, 20 and 21) hybridize to parts of other chromosomes (NAM 1q distal, 4q proximal, 1q proximal and 5q distal, respectively). The other 11 autosomal probes show more than one signal in NAM, with nine (HME 1, 6, 7, 8, (9,10), 11, 14, (16,17) and 23) hybridizing to two chromosomes each; HME 5 and (13,22) show signals in three chromosomes each. Four NAM pairs show association of syntenic blocks to multiple HME probes (Fig. 3, Table 2).

### Chromosomal phylogeny

The comparative analysis of 15 karyotypes from 14 species hybridized with HME whole-chromosome probes [10] generated 56 non-additive multi-state chromosomal characters (Additional file 2: Table S1), which were converted into a matrix (Additional file 3: Table S2) and submitted to parsimony analysis. Characters and character states are detailed in the Additional file 5.

**Fig. 4:**
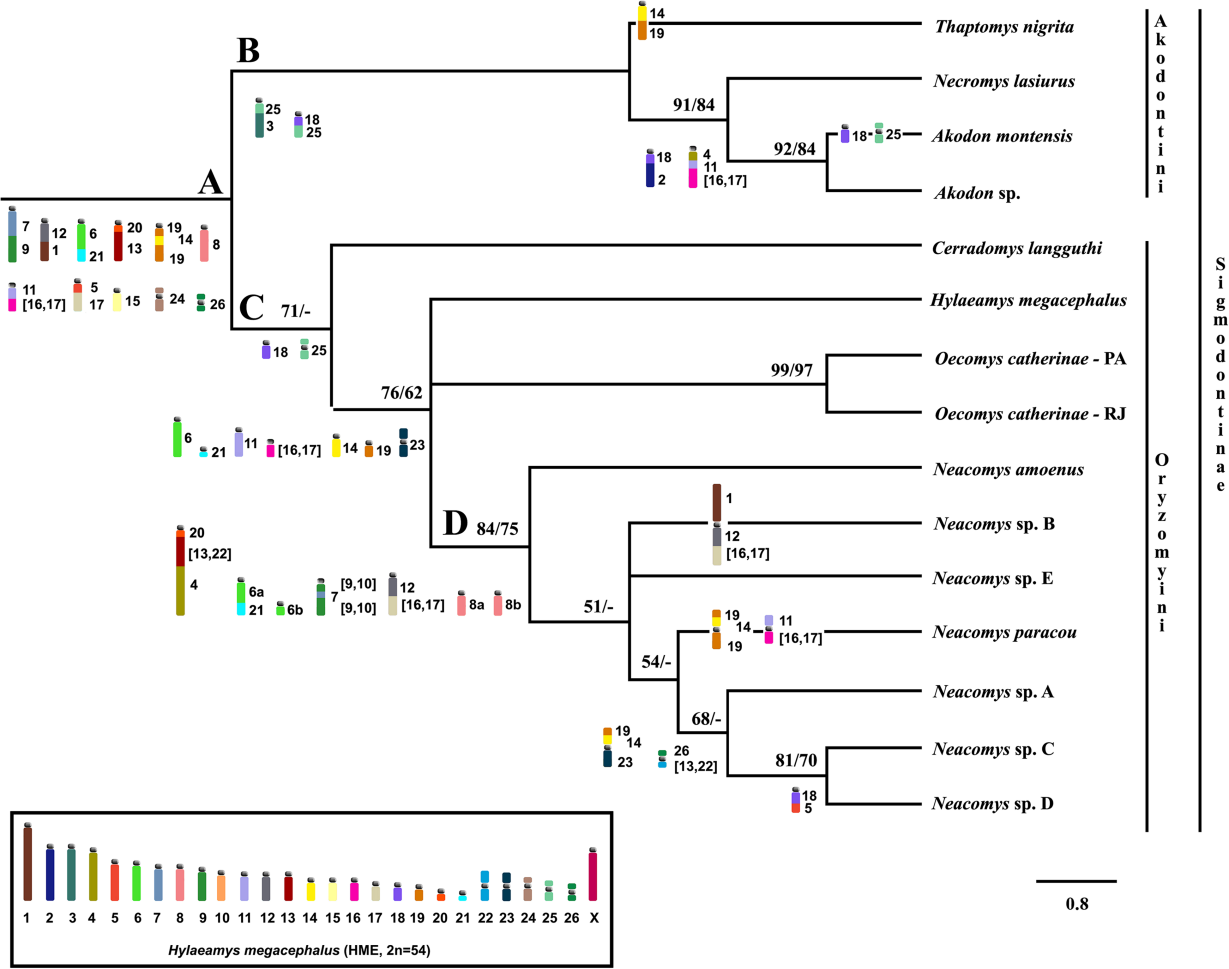
Most parsimonious tree based on matrix of chromosomal characters in TNI, AMO, ASP, NLA (outgroup), HME, CLA, OCA-PA, OCA-RJ, NSP-A, NSP-B, NSP-C, NSP-D, NPA, NSP-E and NAM karyotypes. Karyotype abbreviations as in Table 2. Numbers above branches are maximum parsimony bootstrap values analyzed on PAUP (left) and on T.N.T. (right); below are the chromosomal signatures for each node: Node A (Sigmodontinae subfamily), node B (Akodontini tribe), node C (Oryzomyini tribe), and node D (*Neacomys* genus). The analysis was carried out using PAUP and T.N.T. software packages. Only values above 50% are shown. Inside the box is the ideogram of HME karyotype, as assessed based on HME probes

Both heuristic (PAUP) and exhaustive (T.N.T.) analysis recovered the same phylogenetic relationships with distinct support values (bootstrap; Fig. 4). From PAUP analysis the most parsimonious tree was obtained with consistency index 0.691, retention index 0.764, and homoplasy index 0.308; from T.N.T. analysis the implemented algorithm was FUSE and retrieved a best score of 94 in a single tree from a total of 95, 636, 532 rearrangements. The Sigmodontinae subfamily (Fig. 4, node A) showed an initial branching in two reciprocally monophyletic clades, representing the tribes Akodontini (Fig. 4, node B) and Oryzomyini (Fig. 4, node C), both with good reliability and synapomorphies reinforcing the branches. The monophyly of *Neacomys* was confirmed with bootstrap values of 84% for PAUP analysis and 75% for T.N.T. analysis, and four chromosome signatures (Fig. 4, node D). We recovered a polytomy with no definition among the branches that lead to NSP-B, NSP-E, and the clade composed of NPA, NSP-A, NSP-C and NSP-D. Results are discussed using data retrieved by T.N.T.

## Discussion

### New cytogenetic and distribution data for *Neacomys*

*Neacomys* sp. C and D were first described as “*Neacomys* sp.”, for Marabá and Chaves (Marajó Island) populations, respectively [5]. The samples of *Neacomys* sp. D from Afuá, Marajó Island (Fig. 1, locality 2) present the same karyotype as samples from the Chaves population (2n = 58/FN = 70) [5]. We named the specimens from Marabá (2n = 58/FN = 64) as *Neacomys* sp. C, and those from Afuá/Chaves as *Neacomys* sp. D, to differentiate their karyotypes. The two karyotypes are similar to *Neacomys* sp. A (2n = 58/FN = 68) [7], since they share the same diploid number (2n = 58), with variations in the FN. However, our comparative analyses reveal the differences in sections below (see Complex chromosomal evolution in Neacomys, and on Additional file 4: Table S3 and Additional file 6: Figure S2).

*Neacomys* from Vila Rica (Mato Grosso state, Brazil) has a new cytotype (2n = 58/FN = 66) [8]. The molecular phylogeny (Cytb sequences) [8] showed that the Vila Rica samples belonged to the “Marabá clade”, that corresponds to samples of NSP-C, and proposed that the differences between the two FN (66 and 64) are due to pericentric inversions.

We also described a new cytotype with a different FN for *N. paracou* (2n = 56/FN = 64), distinct from those defined before (2n = 56/FN = 62, 66) [5]. The three karyotypes of *N. paracou* differ by the number of one-armed and bi-armed chromosomes, probably due to two pericentric inversions. Our samples of *N. paracou* were collected in Mazagão (Amapá state, Brazil; Fig. 1, locality 4), which is outside the known distribution for *N. paracou* [3], thus extending its distribution to the southeast of Amapá state.

Recently, a taxonomic revision of *Neacomys spinosus* (2n = 64/FN = 68) was carried out using molecular (Cytb) and morphological data [6]. Three species were recognized: *N. spinosus* (sensu *strictu*), *N. amoenus*, and *N. vargasllosai*. Among them, only *N. amoenus* occurs in Brazil (from eastern Ecuador and northern Peru to central western Brazil and Bolivia). Since the karyotyped samples of the *N. spinosus* complex were from Brazilian localities [2], the 2n = 64/FN = 68 karyotype must be assigned to *N. amoenus*, which means that there is no cytogenetic data for *N. spinosus* at the present time.

The karyotype of *Neacomys* sp. E (2n = 62/FN = 60) was similar to that described for *N. dubosti* (2n = 62) in a previous work that provided the diploid number but not showing the karyotype for this taxon [3]. Another karyotype for *N. dubosti* (2n = 64/FN = 68) was also described in the literature [5]. Comparative analysis of classic banding patterns revealed that the difference between *N. dubosti* [5] and *Neacomys* sp. E is probably due to fusion/fission events. Although the two are karyotypically similar our sample was collected in Santa Bárbara (Pará state, Brazil; Fig. 1, locality 5), while the known distribution for *N. dubosti* is Suriname, French Guyana and north of Amapá state (Brazil) [3–5]. Moreover, our taxonomic identification showed that NSP-E does not correspond to any other valid species described to date for *Neacomys*. Thus, the karyotypic similarity may reflect a convergent evolutionary process, or these taxa may share a common ancestor that diverged recently.

A better delineation of the taxonomic status, distribution and diversity will be possible only after a taxonomic review of members of the genus, including samples from eastern Amazonia.

### Complex chromosomal evolution in *Neacomys*

The comparative chromosome painting analyses among NSP-A (2n = 54/FN = 66), NSP-B (2n = 58/FN = 68) [7], NSP-C (2n = 58/FN = 64), NSP-D (2n = 58/FN = 70), NPA (2n = 56/FN = 64), NSP-E (2n = 62/FN = 60), and NAM (2n = 64/FN = 68) karyotypes allowed us to track the distribution of homologous regions and determine the chromosomal evolution of this group. These analyses show that the chromosomal diversity found is due to 17 fusion/fission events and 1 translocation, pericentric inversions on four syntenic blocks, and six syntenic autosomal blocks with CH amplification/deletion, plus the X chromosome (Additional file 4: Table S3; Additional file 6: Figure S2).

Only four syntenic blocks were found to be stable, with no detectable rearrangement among species of *Neacomys* (HME 8, HME 6, HME 15, and HME 24). Of them, blocks HME 15 and HME 24 were preserved in other karyotypes from the tribes Akodontini and Oryzomyini (with the exception of AMO, ASP and NLA), while HME 8 and HME 6 were involved in distinct rearrangements in different species.

The data above corroborate the evolutionary pattern described for rodents [25], with fusion/fission events as the most common chromosomal rearrangements, followed by pericentric inversions, and these events are considered as a well-established mode of rapid speciation. The roles of different chromosomal rearrangements in the speciation process have been discussed in the literature since the comparison of rat and mouse genomes [25]. These studies revealed that there can be numerous inversions inside conserved blocks, suggesting that these events may have played a leading role in the genomic reorganization of rodents [25].

Although variation in the number of CH blocks was found in *Neacomys* karyotypes, which present bi-armed chromosomes with entirely heterochromatic arms (e.g., NSP-A, NSP-C and NSP-D), this is a frequent event in rodents and is the main cause of the variability of sex chromosome morphology [26, 27]. There is no evidence that such variation is involved in speciation [28], since this kind of structural change does not act as a postzygotic isolating mechanism, or generate deleterious meiotic products that could reduce fertility. Thus, our findings are consistent with the idea that the karyotypes obtained from the Marabá and Marajó Island populations (NSP-C and NSP-D, respectively) belong to the same species and exhibit regional chromosome variation [5]. This high karyotypic reorganization is characteristic of Myomorpha rodents [26] with a few exceptions [29].

We note that the rearrangements among the seven karyotypes from six species of *Neacomys* are not randomly distributed. In fact, Bailey et al. [30] proposed that there are hotspots for chromosomal rearrangements that usually involve repetitive sequences grouped in heterochromatin. The probes HME 5, 11, 14, 19, 23 and 26 are involved in almost half (NSP-A, NSP-C, NSP-D, NPA) or more than half (NSP-B, NSP-E, NAM) of the chromosomes of *Neacomys* karyotypes, in many complex rearrangements like fusion/fission, translocation, CH amplification/deletion and pericentric inversion (Additional file 4: Table S3. Additional file 6: Figure S2).

Despite the karyotypic similarity among *N. minutus* (2n = 35–36/FN = 40), *N. musseri* (2n = 34/FN = 64–68) [2], *N. rosalindae* (2n = 48/FN = 50) and *N. macedoruizi* (2n = 28/FN = 36) [9], we cannot assume that the members of this low-2n group must share chromosomal associations with each other, or with other species of *Neacomys*.

Our chromosome painting analysis of two species of *Proechimys* with comparable 2n and karyotypes composed mostly of bi-armed chromosomes (*P. roberti* with 2n = 30/FN = 54 and *P. goeldii* with 2n = 24♀25♂/FN = 42) suggest that multiple translocations largely account for the karyotypic difference, with full preservation of only three whole chromosomes [31]. Thus, an investigation of the genomes of *N. minutus*, *N. musseri* [2], *N. rosalindae* and *N. macedoruizi* [9] could help reveal whether the signatures detected in the present study (see Phylogenetic relationships and chromosomal signatures) are maintained in these taxa despite the drastic 2n decrease, or whether they are exclusive for the high-2n group (from 54 to 64). The consolidation of this data could improve our understanding of the chromosomal evolution of *Neacomys* and potentially facilitate the reconstruction of its hypothetical ancestral karyotype.

Moreover, given that the resolution of chromosome painting is limited and peri/paracentric inversions and reciprocal translocations are difficult to detect, the chromosome evolution in *Neacomys* may be more complex than currently thought, and could involve more events than those identified in the present study.

### Phylogenetic relationships and chromosomal signatures

The phylogenetic analysis using chromosomal data reflects the molecular relationships of the Akodontini and Oryzomyini tribes [32] (Fig. 4). We corroborate the ancestral traits previously proposed for the Sigmodontinae subfamily (Node A, Fig. 4) [7, 23]: HME 7/(9,10), 1/12, 6/21, 11/(16,17), 5/(16,17), 15, 24 and 26. We added HME 8, since it is shared by all Akodontini, as well as HME and OCA (Oryzomyini).

Concerning the Akodontini tribe, TNI was the sister to the other species, followed by NLA, and a clade was comprised of the species of *Akodon* (AMO + ASP). All members of the Akodontini tribe exhibited the synapomorphies HME 3/25 and 18/25 (Node B, Fig. 4). TNI presented the HME 14/19 association as an autapomorphy, while the clade formed by NLA and AMO + ASP preserved the syntenic blocks, HME 4/11/(16,17) and 2/18, which are chromosomal signatures for this clade.

The relationships within the clade formed by the Oryzomyini species are similar to those recovered in the molecular analysis [32], and all Oryzomyini species presented the signatures previously proposed [7]: HME 8a, 8b, 18, 25 (Node C, Fig. 4). The main difference is related to the position of NPA: In the molecular analysis [7] NPA is the sister species for all *Neacomys*, whereas in the cytogenetics phylogeny, this position is occupied by NAM and NPA is the sister species only of the clade “NSP-A and NSP-C+NSP-D”. This difference may be consequence of the sampling, since NPA clearly exhibits high genetic variability [13]. This species occupies a large geographic distribution and varies in its haplotypes [13] and chromosomes [5]. Better sampling of NPA, on both the molecular and karyotypic levels, may allow researchers to resolve this discrepancy. Alternatively, there may have been a Long Branch Attraction (LBA) involving NPS-E and NPS-B, since they show rapid karyotypic evolution.

The chromosomal topology analysis recovered the monophyly of *Neacomys*, with all species included in a polytomy. We compared our chromosomal topology with the previous molecular phylogeny (Cytb and COI) [7], which had included the three undescribed species (NSP-A, NSP-B and NSP-C + NSP-D).

Although the lack of more phylogenetic signals due to the great similarity among *Neacomys* karyotypes, and the moderate resolution of the chromosomal tree, both topologies (molecular and chromosomal) recovered the clade “NSP-A and NSP-C+NSP-D” as the most recent divergent branch, supported by the signatures HME 19/14/23 and 26/(13,22). All *Neacomys* karyotypes exhibit the synapomorphic signatures (Node D, Fig. 4) proposed [7]: (HME 20/(13,22)/4, 6a/21, (9,10)/7/(9,10) and 12/(16,17)). The entirety of HME 8 is an ancestral trait in Sigmodontinae (Node A, Fig. 4), where it corresponds to MMU 9 [23]. In *Neacomys*, however, HME 8 is split into two blocks, 8a and 8b. This is also found in CLA, probably reflecting a homoplasy. HME 6/21 is also an ancestral trait in Sigmodontinae (Node A, Fig. 4), where it corresponds to MMU 2 [23]. In *Neacomys* there was a fission in the HME 6 portion, originating HME 6a/21 and HME 6b as an independent pair.

Concerning the phylogenetic position of species of *Neacomys* in the molecular topology [7] and the chromosomal signatures, we noted that NPA presents two Sigmodontinae plesiomorphic characters (HME 11/16 and 19/14/19); however, there are alterations in the chromosomal morphology of both associations (probably due to inversions or centromeric repositioning, which are absent in the other karyotypes of the genus. However, NSP-A and NSP-C + NSP-D exhibit a different chromosome form (HME 19/14/23). We propose that this signature (HME 19/14/19) is present in the ancestral species of the genus, maintained in NPA, derived in NSP-A, NSP-C, and NSP-D (HME 19/14/23), while it was lost in intermediary branches (NAM, NSP-B, and NSP-E).

Another ancestral trait observed is the chromosomal association HME 1/12, present as a derived form in NSP-B (HME 1/12/(16,17)), while the other karyotypes of *Neacomys* exhibit HME 12/(16,17). The karyotype of this group was shaped by multiple fusion/fission events that occurred during its evolution [5]. Thus, HME 1/12/(16,17) was probably present in the ancestral karyotype of *Neacomys*, with the HME 1 segment becoming dissociated from the syntenic block during diversification events of the genus, and being maintained only in NSP-B (2n = 54). NSP-A and NSP-C + NSP-D exhibit one ancestral trait for the Sigmodontinae (HME 26) that is changed by the fusion (HME (13,22)/26), which is a signature for these two species.

In conclusion, based on the classic banding and comparative chromosome painting results, we observe that the karyotypic divergence among species of *Neacomys* followed the phylogenetic relationship, as NPA retained some ancestral traits from the subfamily, while NSP-A, NSP-B and NSP-C + NSP-D shared other synapomorphic characters. This pattern was also observed in many taxonomic groups, with a few exceptions [16].

## Conclusions

We describe new karyotypes and distribution areas for species of *Neacomys* from eastern Amazon and detect, through chromosome painting, that multiple fusion/fission events are the main cause of the karyotypic diversity in this group with high diploid numbers, followed by pericentric inversions and amplification/deletion of CH. Besides, our chromosomal phylogeny recovered the monophyly of *Neacomys*, and demonstrated the synapomorphies of the genus (HME 6a/21, 6b, 8a, 8b, (9,10)/7b/(9,10), 12/(16,17), 20/(13,22)/4).

## Methods

### Sampling

In a previous publication [5] we described the classic cytogenetic bandings for two new karyotypes from a *Neacomys* taxon that we called “*Neacomys* sp.”. In the present work we made chromosome painting in this taxon. However, as the two karyotypes are different (see Results and Discussion), here we are calling them “*Neacomys* sp. C” and “*Neacomys* sp. D”. We studied the karyotypes of five *Neacomys* sp. C (three males and two females) from Marabá, Pará state, Brazil (Fig. 1, locality 1); 10 *Neacomys* sp. D, five of which (three males and two females) from Chaves town, Marajo island, Para state, Brazil (Fig. 1, locality 3), and five (three males and two females) from Afuá town (Fig. 1, locality 2), a different locality in Marajo Island that had not sampled before. Also we studied three specimens of *N. paracou* (two males and one female) from Mazagão municipality (Fig. 1, locality 4), Amapá state, Brazil; one specimen (male) of *Neacomys* sp. E from Santa Bárbara municipality (Fig. 1, locality 5), Pará state, Brazil; and six specimens of *N. amoenus* (two males and four females) from Alta Floresta municipality (Fig. 1, locality 6), Mato Grosso state, Brazil.

The samples collections were made using pitfall traps [33]. Pitfall trap lines were distributed in three plots of each locality (Table 1, Fig. 1). In each plot, two traps lines were set up using six 60-l buckets set at intervals of 10 m. The traps were checked early in the morning for eight consecutive days. The specimens were deposited at the zoological collections of Museu de Zoologia da Universidade Federal do Pará (UFPA), Belém, Pará; Museu Paraense Emílio Goeldi (MPEG), Belém, Pará; Laboratório de Mamíferos, Instituto de Pesquisas Científicas e Tecnológicas do Estado do Amapá (IEPA), Macapá, Amapá; and Universidade Federal de Mato Grosso (UFMT), Cuiabá, Mato Grosso. All institutions are located in Brazil.

### Cytogenetics

The metaphasic chromosomal samples were acquired from bone marrow extraction [34] and by fibroblast cell culture made from skin biopsy, established at Centro de Estudos Avançados da Biodiversidade, Laboratório de Citogenética (ICB, UFPa, Brazil). G-banding was made following Sumner et al. [35], and C-banding was performed following Sumner [36]. C-banding was performed on G-banded metaphases for the correct chromosomal assignment. Whole chromosome probes of HME [11] were used for FISH studies, with three of the 24 HME probes corresponding to two chromosome pairs each (HME (9,10), (13,22), and (16,17)). We used sequential DAPI staining and converted the DAPI staining in G-band pattern for the correct assignment of the hybridized chromosomes. Chromosome classification was established according to Levan et al. [37]. Digital images were obtained by Nis-Elements software and Nikon H550S microscopy. The final edition of images was made on Adobe Photoshop CS6.

### Phylogenetic analysis

The Maximum Parsimony (MP) phylogenetic analysis was made using PAUP program (Phylogenetic Analysis Using Parsimony) version 4.0a159 [38] and T.N.T. (“Tree analyses Using New Technologies”) version 1.1 [39]. Branch support values were calculated with 1000 bootstrap replicates for both analyses. The heuristic search was made using Tree Bisection Reconnection (TBR) in PAUP. An exhaustive search was made using T.N.T.; the Fuse algorithmic was used to examine a total of 95,636,532 rearrangements. A list of characters and character states was created and converted into a non-additive (unordered) multi-state character matrix on Mesquite program version 3.10 [40]. The chosen non-additive multi-state characters were chromosomal morphology, number and syntenic blocks based on HME probes hybridized to metaphases of *Neacomys* sp. C (NSP-C), *Neacomys* sp. D (NSP-D), *Neacomys* sp. E (NSP-E), *N. paracou* (NPA), *N. amoenus* (NAM) (present study), and comparing with species from previous studies: *Neacomys* sp. A (NSP-A), *Neacomys* sp. B (NSP-B) [7], *Cerradomys langguthi* (CLA) [11], *Thaptomys nigrita* (TNI), *Akodon montensis* (AMO) [22], *Akodon* sp. (ASP), *Necromys lasiurus* (NLA) [23], *Oecomys catherinae*-Pará (OCA-PA), and *O. catherinae*-Rio de Janeiro (OCA-RJ) [24].

We did not put weight on the characters since we cannot precisely determine the probability of each rearrangement. Also, the characters are used as qualitative traits and not quantitative, as they are rare events with strong phylogenetic signals [41]. The karyotypes of TNI, AMO [22], ASP and NLA [23] were used as outgroups since they belong to the Akodontini tribe, a sister taxon to the Oryzomyini tribe, where *Neacomys* and the other genera belong. Both tribes belong to the Sigmodontinae subfamily. The tree was displayed and edited in Figtree program version 1.4.2 (http://tree.bio.ed.ac.uk/software/figtree/).

## Supplementary information

**Additional file 1: Figure S1.** C-banding in *Neacomys*. (A) *Neacomys* sp. D (NSP-D, 2n = 58/FN = 70); (B) *N. paracou* (NPA, 2n = 56/FN = 64); (C) *Neacomys* sp. E (NSP-E, 2n = 62/FN = 60); (D) *N. amoenus* (NAM, 2n = 64/FN = 68).

**Additional file 2: Table S1.** Non-additive multi-state character list. Character descriptions are detailed in Additional file 5.

**Additional file 3: Table S2.** Chromosome character data matrix of non-additive multi-state character employed in the Maximum Parsimony analysis.

**Additional file 4: Table S3.** Chromosomal rearrangements among seven karyotypes of six Neacomys species, based on chromosome painting with Hylaeamys megacephalus probes (HME) [10].

**Additional file 5.** Analysis and description of characters and characters states.

**Additional file 6: Figure S2.** Ideograms showing the rearrangements among the karyotypes of six species of *Neacomys*, as assessed based on *Hylaeamys megacephalus* whole chromosome probes [11]. (H) Indicates large block of constitutive heterochromatin. Karyotype abbreviations as in Table 2.

## Data Availability

All the data supporting findings are contained within the manuscript and in the supplemental files. If any additional information is necessary, please contact the corresponding author.

## Abbreviations

2n: Diploid number
AMO: *Akodon montensis*
ASP: *Akodon* sp.
CH: Constitutive heterochromatin
CLA: *Cerradomys langguthi*
COI: Cytochrome C Oxidase – subunit I
Cytb: Cytochrome B
FISH: Fluorescent In-Situ Hybridization
FN: Fundamental number
HME: *Hylaeamys megacephalus*
IEPA: Laboratório de Mamíferos, Instituto de Pesquisas Científicas e Tecnológicas do Estado do Amapá
IRBP: Interphotoreceptor Retinoid Binding Protein
MP: Maximum Parsimony
MPEG: Museu Paraense Emílio Goeldi
NAM: *Neacomys amoenus*
NLA: *Necromys lasiurus*
NPA: *Neacomys* *paracou*
NSP-A: *Neacomys* sp. A
NSP-B: *Neacomys* sp. B
NSP-C: *Neacomys* sp. C
NSP-D: *Neacomys* sp. D
NSP-E: *Neacomys* sp. E
OCA-PA: *Oecomys catherinae*-Pará
OCA-RJ: *Oecomys* *catherinae*-Rio de Janeiro
PAUP: Phylogenetic Analysis Using Parsimony
rDNA: Ribosomal DNA
T.N.T.: Tree Analyses Using New Technologies
TBR: Tree Bisection Reconnection
TNI: *Thaptomys nigrita*
UFMT: Universidade Federal de Mato Grosso
UFPA: Museu de Zoologia da Universidade Federal do Pará

## References

[1] Baker RJ, Koop BF, Haiduk MW (1983). Resolving systematic relationships with G-bands. A study of five genera of South American Cricetine rodents. Syst Zool.

[2] Patton JL, Silva MN, Malcolm JR (2000). Mammals of the Rio Juruá and the evolutionary and ecological diversification of Amazonia. Bul Am Mus Nat Hist.

[3] Voss RS, Lunde DP, Simmons NB (2000). The mammals of Paracou, French Guiana: a neotropical lowland rainforest fauna. Part 2. Nonvolant species. Bull Am Mus Nat Hist.

[4] Redi C. A., Zacharias H., Merani S., Oliveira-Miranda M., Aguilera M., Zuccotti M., Garagna S., Capanna E. (2005). Genome Sizes in Afrotheria, Xenarthra, Euarchontoglires, and Laurasiatheria. Journal of Heredity.

[5] da Silva WO, Pieczarka JC, Rossi RV, Schneider H, Sampaio I, Miranda CL, et al. Diversity and karyotypic evolution in the genus *Neacomys* (Rodentia, Sigmodontinae). Cytogenet Genome Res. 2015. 10.1159/000441173. 10.1159/000441173 26587770

[6] HURTADO NATALI, PACHECO VÍCTOR (2017). Revision of Neacomys spinosus (Thomas, 1882) (Rodentia: Cricetidae) with emphasis on Peruvian populations and the description of a new species. Zootaxa.

[7] Oliveira Da Silva Willam, Pieczarka Julio Cesar, Ferguson-Smith Malcolm Andrew, O’Brien Patricia Caroline Mary, Mendes-Oliveira Ana Cristina, Sampaio Iracilda, Carneiro Jeferson, Nagamachi Cleusa Yoshiko (2017). Chromosomal diversity and molecular divergence among three undescribed species of Neacomys (Rodentia, Sigmodontinae) separated by Amazonian rivers. PLOS ONE.

[8] Di-Nizo Camilla Bruno, Banci Karina Rodrigues da Silva, Sato-Kuwabara Yukie, Silva Maria José de J. (2017). Advances in cytogenetics of Brazilian rodents: cytotaxonomy, chromosome evolution and new karyotypic data. Comparative Cytogenetics.

[9] Sánchez-Vendizú Pamela, Pacheco Víctor, Vivas-Ruiz Dan (2018). An Introduction to the Systematics of Small-Bodied Neacomys (Rodentia: Cricetidae) from Peru with Descriptions of Two New Species. American Museum Novitates.

[10] Hice CL, Velazco PM (2012). The non-volant mammals of the Reserva Nacional Allpahuayo Mishana, Iquitos, Peru. Special Publications. Mus Texas Tech Univ.

[11] Nagamachi Cleusa Yoshiko, Pieczarka Julio Cesar, O’Brien Patricia Caroline Mary, Pinto Jamilly Amaral, Malcher Stella Miranda, Pereira Adenilson Leão, das Dores Rissino Jorge, Mendes-Oliveira Ana Cristina, Rossi Rogério Vieira, Ferguson-Smith Malcolm Andrew (2013). FISH with whole chromosome and telomeric probes demonstrates huge karyotypic reorganization with ITS between two species of Oryzomyini (Sigmodontinae, Rodentia): Hylaeamys megacephalus probes on Cerradomys langguthi karyotype. Chromosome Research.

[12] Weksler M, Percequillo AR, Voss RS (2006). Ten new genera of Oryzomyine rodents (Cricetidae, Sigmodontinae). Am Mus Novitates.

[13] Catzeflis F, Tilak M. Molecular systematic of neotropical spiny mice (*Neacomys*: Sigmodontinae, Rodentia) from the Guiana region. Mammalia. 2009. 10.1515/MAMM.2009.037.

[14] Percequillo AR, Weksler M, Costa LP (2011). A new genus and species of rodent from the Brazilian Atlantic Forest (Rodentia: Cricetidae: Sigmodontinae: Oryzomyini), with comments on oryzomyine biogeography. Zool J Linn Soc Lond.

[15] Ventura K, O’Brien PC, Yonenaga-Yassuda Y, Ferguson-Smith MA (2009). Chromosome homologies of the highly rearranged karyotypes of four Akodon species (Rodentia, Cricetidae) resolved by reciprocal chromosome painting: the evolution of the lowest diploid number in rodents. Chromosom Res.

[16] Graphodatsky A., Ferguson-Smith M.A., Stanyon R. (2012). A Short Introduction to Cytogenetic Studies in Mammals with Reference to the Present Volume. Cytogenetic and Genome Research.

[17] Ao L, Mao X, Nie W, Gu X, Feng Q, Wang J, et al. Karyotypic evolution and phylogenetic relationships in the order Chiroptera as revealed by G-banding comparison and chromosome painting. Chromosom Res. 2007. 10.1007/s10577-007-1120-7. 10.1007/s10577-007-1120-7 17310301

[18] Pieczarka J. C., Gomes A. J. B., Nagamachi C. Y., Rocha D. C. C., Rissino J. D., O’Brien P. C. M., Yang F., Ferguson-Smith M. A. (2013). A phylogenetic analysis using multidirectional chromosome painting of three species (Uroderma magnirostrum, U. bilobatum and Artibeus obscurus) of subfamily Stenodermatinae (Chiroptera-Phyllostomidae). Chromosome Research.

[19] Ribas Talita Fernanda Augusto, Rodrigues Luis Reginaldo Ribeiro, Nagamachi Cleusa Yoshiko, Gomes Anderson José Baia, Rissino Jorge das Dores, O'Brien Patricia Caroline Mary, Yang Fengtang, Ferguson-Smith Malcolm Andrew, Pieczarka Julio Cesar (2015). Phylogenetic Reconstruction by Cross-Species Chromosome Painting and G-Banding in Four Species of Phyllostomini Tribe (Chiroptera, Phyllostomidae) in the Brazilian Amazon: An Independent Evidence for Monophyly. PLOS ONE.

[20] Gomes AJB, Nagamachi CY, Rodrigues LRR, Benathar TCM, Ribas TFA, O’Brien PCM, et al. Chromosomal phylogeny of Vampyressine bats (Chiroptera, Phyllostomidae) with description of two new sex chromosome systems. BMC Evol Biol. 2016. 10.1186/s12862-016-0689-x. 10.1186/s12862-016-0689-x PMC4893233 27260645

[21] Hass Iris, Sbalqueiro Ives José, Müller Stefan (2008). Chromosomal phylogeny of four Akodontini species (Rodentia, Cricetidae) from Southern Brazil established by Zoo-FISH using Mus musculus (Muridae) painting probes. Chromosome Research.

[22] Suárez Pablo, Nagamachi Cleusa Yoshiko, Lanzone Cecilia, Malleret Matias Maximiliano, O’Brien Patricia Caroline Mary, Ferguson-Smith Malcolm Andrew, Pieczarka Julio Cesar (2015). Clues on Syntenic Relationship among Some Species of Oryzomyini and Akodontini Tribes (Rodentia: Sigmodontinae). PLOS ONE.

[23] Pereira Adenilson Leão, Malcher Stella Miranda, Nagamachi Cleusa Yoshiko, O’Brien Patricia Caroline Mary, Ferguson-Smith Malcolm Andrew, Mendes-Oliveira Ana Cristina, Pieczarka Julio Cesar (2016). Extensive Chromosomal Reorganization in the Evolution of New World Muroid Rodents (Cricetidae, Sigmodontinae): Searching for Ancestral Phylogenetic Traits. PLOS ONE.

[24] Malcher Stella Miranda, Pieczarka Julio Cesar, Geise Lena, Rossi Rogério Vieira, Pereira Adenilson Leão, O’Brien Patricia Caroline Mary, Asfora Paulo Henrique, Fonsêca da Silva Victor, Sampaio Maria Iracilda, Ferguson-Smith Malcolm Andrew, Nagamachi Cleusa Yoshiko (2017). Oecomys catherinae (Sigmodontinae, Cricetidae): Evidence for chromosomal speciation?. PLOS ONE.

[25] Romanenko S.A., Volobouev V. (2012). Non-Sciuromorph Rodent Karyotypes in Evolution. Cytogenetic and Genome Research.

[26] Romanenko S A, Perelman P L, Trifonov V A, Graphodatsky A S (2011). Chromosomal evolution in Rodentia. Heredity.

[27] Kasahara S. Introdução à Pesquisa em Citogenética de Vertebrados. 1st ed: Ribeirão Preto, SP, Brasil. Sociedade Brasileira de Genética; 2009.

[28] King M (1987). Chromosomal rearrangements, speciation and the theoretical approach. Heredity.

[29] Swier VJ, Bradley RD, Rens W, Elder FFB, Baker RJ. Patterns of chromosomal evolution in Sigmodon, evidence from whole chromosome paints. Cytogenet Genome Res. 2009. 10.1159/000218747. 10.1159/000218747 PMC2918645 19617697

[30] Bailey JA, Baertsch R, Kent WJ, Haussler D, Eichler EE (2004). Hotspots of mammalian chromosomal evolution. Genome Biol.

[31] Da Silva WO, Rodrigues da Costa MJ, Pieczarka JC, Rissino J, Pereira JC, Ferguson-Smith MA, Nagamachi CY. Identification of two independent X-autosome translocations in closely related mammalian (Proechimys) species. Sci Rep. 2019. 10.1038/s41598-019-40593-8. 10.1038/s41598-019-40593-8 PMC6411977 30858413

[32] Pardiñas UFJ, Teta P, Salazar-Bravo J (2015). A new tribe of Sigmodontinae rodents (Cricetidae). Mastozool Neotrop.

[33] Corn PS, Heyer WR, Donnelly MA, McDiarmid RW, Hayek LC, Foster MS (1994). Straight-line drift fences and pitfall traps. Measuring and monitoring biological standard methods for amphibians.

[34] Ford CE, Hamerton JL (1956). A colchicine, hypotonic – citrate, squash sequence for mammalian chromosomes. Stain Technol.

[35] Sumner AT, Evans HJ, Buckland RA (1971). New technique for distinguishing between human chromosomes. Nature (Lond) New Biol.

[36] Sumner AT (1972). A simple technique for demonstrating centromeric heterochromatin. Exp Cell Res.

[37] Levan A, Fredga K, Sandberg AA (1964). Nomenclature for centromeric position on chromosomes. Hereditas.

[38] Swofford DL (2002). PAUP*. Phylogenetic analysis using parsimony (*and other methods). Version 4.

[39] Goloboff PA, Farris JS, Nixon KC (2008). T.N.T., a free program for phylogenetic analysis. Cladistics.

[40] Maddison WP, Maddison DR (2018). Mesquite: a modular system for evolutionary analysis. Version 3.40.

[41] Rokas A, Holland PWH (2000). Rare genomic changes as a tool for phylogenetics. Trends Ecol Evol.

