# Supplementary figures and images for: Chromosomal phylogeny and comparative chromosome painting among Neacomys species (Rodentia, Sigmodontinae) from eastern Amazonia
Source: BMC Evol Biol. 2019 Oct 10;19:184. doi: 10.1186/s12862-019-1515-z (PMC6785907; doi:10.1186/s12862-019-1515-z)

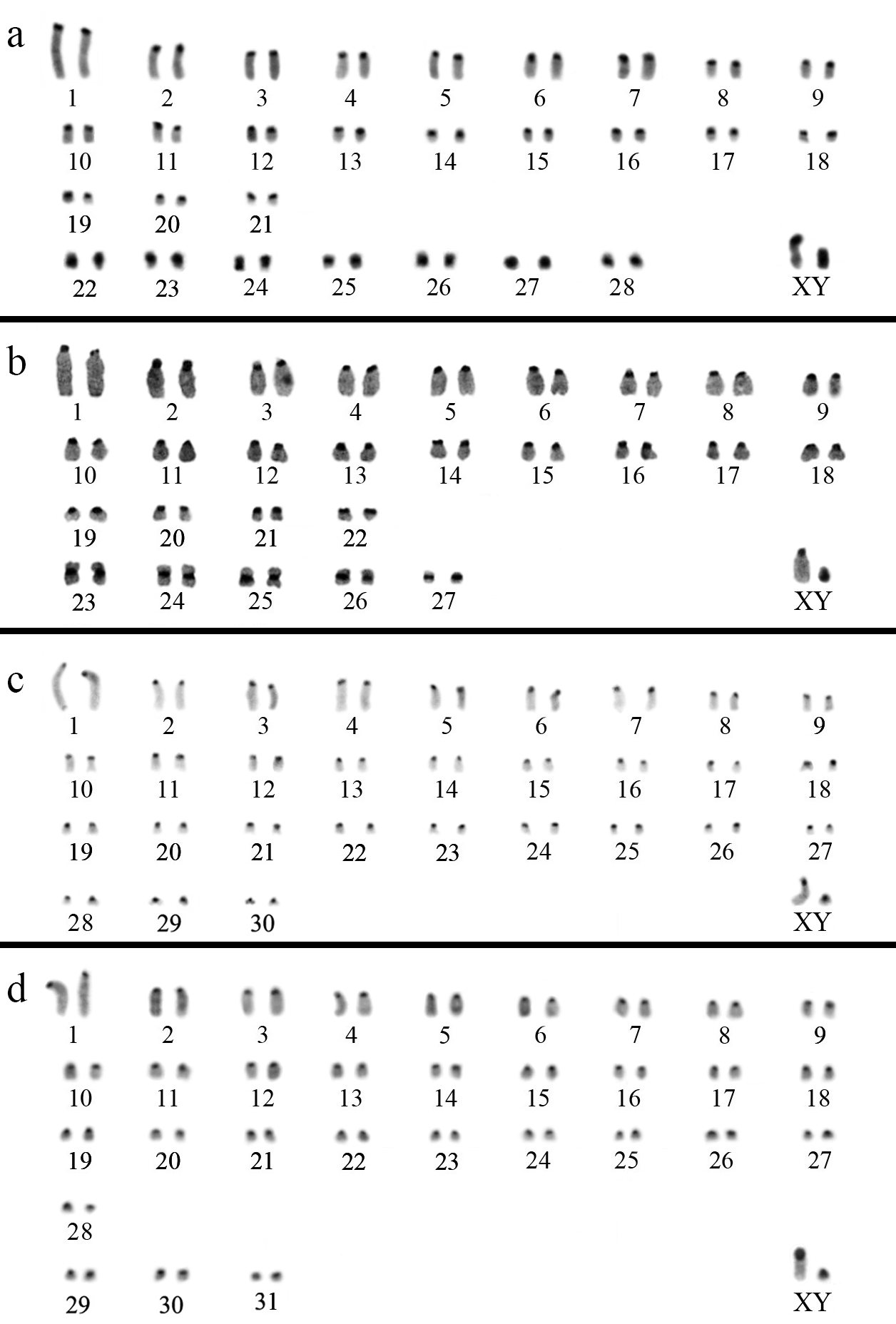

Supplement: Supplementary file 1 — Additional file 1: Figure S1. C-banding in Neacomys. (A) Neacomys sp. D (NSP-D, 2n = 58/FN = 70); (B) N. paracou (NPA, 2n = 56/FN = 64); (C) Neacomys sp. E (NSP-E, 2n = 62/FN = 60); (D) N. amoenus (NAM, 2n = 64/FN = 68). [file 12862_2019_1515_MOESM1_ESM.tif]

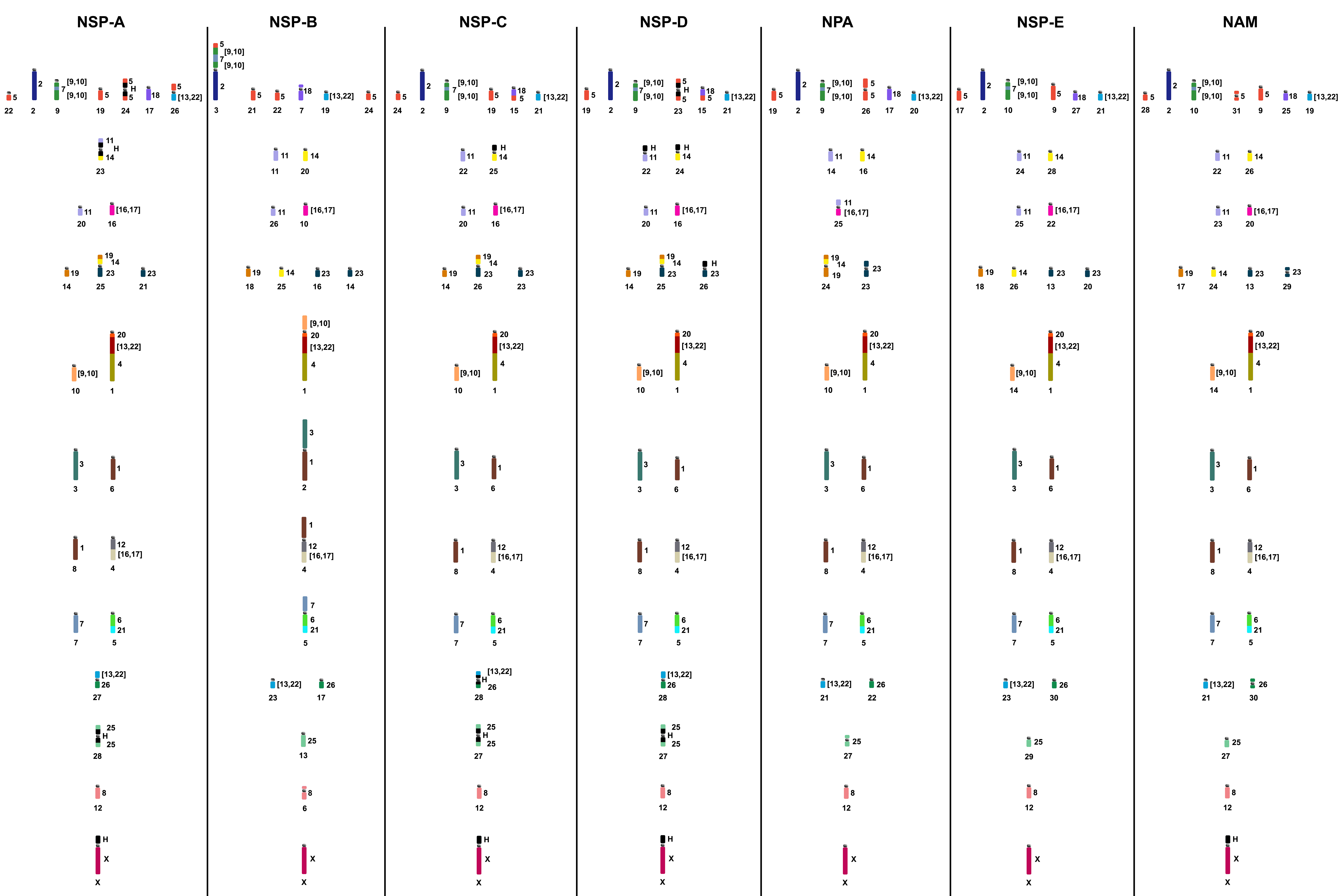

Supplement: Supplementary file 6 — Additional file 6: Figure S2. Ideograms showing the rearrangements among the karyotypes of six species of Neacomys, as assessed based on Hylaeamys megacephalus whole chromosome probes [11]. (H) Indicates large block of constitutive heterochromatin. Karyotype abbreviations as in Table 2. [file 12862_2019_1515_MOESM6_ESM.jpg]
