# Supplementary material for: Chromosomal phylogeny and comparative chromosome painting among Neacomys species (Rodentia, Sigmodontinae) from eastern Amazonia
Source: BMC Evol Biol. 2019 Oct 10;19:184. doi: 10.1186/s12862-019-1515-z (PMC6785907; doi:10.1186/s12862-019-1515-z)
Supplement: Supplementary file 3 — Additional file 3: Table S2. Chromosome character data matrix of non-additive multi-state character employed in the Maximum Parsimony analysis. [file 12862_2019_1515_MOESM3_ESM.docx]

**Table S2** **Chromosome character data matrix of non-additive multi-state character employed in the Maximum Parsimony analysis.** Karyotype abbreviations as in Table 2. Characters are detailed in Table 3. Non-applicable data (-).

| **Taxon/Character** | **1-56** |
| --- | --- |
| **TNI** | 1101200011 101010-000 0001100010 00010-0110 1010000000 000010 |
| **AMO** | 1101201011 1110-0-011 1111011101 0001011110 1001010101 000001 |
| **ASP** | 1102301011 2110-1-011 1111011101 01010-1010 1001010001 000111 |
| **NLA** | 1102201011 1110-1-010 0101101011 0101010010 1001010000 000010 |
| **HME** | 0000000000 0000000000 0000000000 00000-0000 0000000000 000000 |
| **CLA** | 1211200111 0200101110 0000000000 0001000010 1000110100 000100 |
| **OCA-PA** | 2000220011 0111101-00 0000000000 00100-0000 1000000010 011000 |
| **OCA-RJ** | 2100210011 0111101-00 0000000000 00100-0000 1000000010 011000 |
| **NSP-A** | 1000311111 0111100-00 0000000001 0000120001 1100001000 100000 |
| **NSP-B** | 1000311111 0011101110 0000000000 0000120001 1100000000 000000 |
| **NSP-C** | 1000211111 0111100-00 0000000000 1000120001 1100011000 100000 |
| **NSP-D** | 1000211111 0111100-00 0000000000 1000120001 1100011000 100000 |
| **NPA** | 1000111111 0110100100 0000000000 0000120011 1101010000 000000 |
| **NDU** | 1000111111 0011101100 0000000000 0000120001 1100000000 000000 |
| **NAM** | 1000211111 0011101000 0000000000 0000120001 1100000000 000000 |
