# Supplementary material for: Chromosomal phylogeny and comparative chromosome painting among Neacomys species (Rodentia, Sigmodontinae) from eastern Amazonia
Source: BMC Evol Biol. 2019 Oct 10;19:184. doi: 10.1186/s12862-019-1515-z (PMC6785907; doi:10.1186/s12862-019-1515-z)
Supplement: Supplementary file 4 — Additional file 4: Table S3. Chromosomal rearrangements among seven karyotypes of six Neacomys species, based on chromosome painting with Hylaeamys megacephalus probes (HME) [10]. [file 12862_2019_1515_MOESM4_ESM.docx]

**Table S3 Chromosomal rearrangements among seven karyotypes of six *Neacomys* species, based on chromosome painting with *Hylaeamys megacephalus* probes (HME) [10].** Left column (1) corresponds to syntenic blocks detected on *Neacomys* karyotypes. Right columns (2-8) correspond to homology among different chromosomes. Karyotype abbreviations as in Table 2.

| **Chromosome Rearrangements/Taxa** | **NSP-A** | **NSP-B** | **NSP-C** | **NSP-D** | **NPA** | **NSP-E** | **NAM** |
| --- | --- | --- | --- | --- | --- | --- | --- |
| **Fusion/fission, translocation** |  |  |  |  |  |  |  |
| HME 5 (2-4 segments associated or not) | 19+22+24+26 | 3+21+22+24 | 15+19+24 | 15+19+23 | 19+26 | 9, 17 | 9, 28+31 |
| HME 5, HME (9,10)/7/(9,10), HME 2 | 22, 9, 2 | 3 | -, 9, 2 | -, 9, 2 | -, 9, 2 | -, 10, 2 | -, 10, 2 |
| HME 5, HME (13,22) | 26 | 24, 19 | -, 21 | -, 21 | -, 20 | -, 21 | -, 19 |
| HME 18/5 | - | - | 15 | 15 | - | - | - |
|  |  |  |  |  |  |  |  |
| HME 11, HME 14 | 23 | 11, 20 | 22, 25 | 22, 24 | 14, 16 | 24, 28 | 22, 26 |
| HME 11, HME (16,17) | 20, 16 | 26, 10 | 20, 16 | 20, 16 | 25 | 25, 22 | 23, 20 |
|  |  |  |  |  |  |  |  |
| HME 19, HME 19/14/23, HME 23 | 14, 25, 21 | - | 14, 26, 23 | 14, 25, 26 | - | - | - |
| HME 19, HME 14, HME 23 | - | 18, 25, 16+14 | - | - | - | 18, 26, 13+20 | 17, 14, 13+29 |
| HME 19/14/19, HME 23 | - | - | - | - | 24, 23 | - | - |
|  |  |  |  |  |  |  |  |
| HME (9,10), HME 20/(13,22)/4 | 10, 1 | 1 | 10, 1 | 10, 1 | 10, 1 | 14, 1 | 14, 1 |
| HME 3, HME 1 | 3, 6 | 2 | 3, 6 | 3, 6 | 3, 6 | 3, 6 | 3, 6 |
| HME 1, HME 12/(16,17) | 8, 4 | 4 | 8, 4 | 8, 4 | 8, 4 | 8, 4 | 8, 4 |
| HME 7, HME 6/21 | 7, 5 | 5 | 7, 5 | 7, 5 | 7, 5 | 7, 5 | 7, 5 |
| HME (13,22), HME 26 | 27 | 23, 17 | 28 | 28 | 21, 22 | 23, 30 | 21, 30 |
|  |  |  |  |  |  |  |  |
| **Pericentric inversion** |  |  |  |  |  |  |  |
| HME 8 | 12(oa) | 6(ba) | 12(oa) | 12(oa) | 12(oa) | 12(oa) | 12(oa) |
| HME 18 | 17(oa) | 7(ba) | - | - | 17(oa) | 27(oa) | 25(oa) |
| HME 25 | 28(ba) | 13(oa) | 27(ba) | 27(ba) | 27(ba) | 29(oa) | 27(oa) |
| HME 26 | - | 17(oa) | - | - | 22(oa) | 30(oa) | 30(ba) |
|  |  |  |  |  |  |  |  |
| **Amplification/deletion of constitutive heterochromatin** |  |  |  |  |  |  |  |
| HME 5 | 24(H) | - | - | 23(H) | - | - | - |
| HME 11 | 23(H) | 11 | 22 | 22(H) | 14 | 24 | 22 |
| HME 14 | 23(H) | 20 | 25(H) | 24(H) | 16 | 28 | 26 |
| HME 23 | 21 | 14 | 23 | 26(H) | - | 20 | 29 |
| HME 25 | 28(H) | 13 | 27(H) | 27(H) | 27 | 29 | 27 |
| HME (13,22)/26 | 27 | - | 28(H) | 28 | - | - | - |
| HME X | X(H) | X | X(H) | X(H) | X | X | X(H) |
|  |  |  |  |  |  |  |  |
| **No detectable rearrangements** |  |  |  |  |  |  |  |
| HME 6 | 18 | 8 | 18 | 18 | 18 | 19 | 18 |
| HME 8 | 13 | 15 | 13 | 13 | 13 | 16 | 16 |
| HME 15 | 15 | 9 | 17 | 17 | 15 | 15 | 15 |
| HME 24 | 11 | 12 | 11 | 11 | 11 | 11 | 11 |

**Legend:** One-armed chromosome (oa). Bi-armed chromosome (ba). Presence of large block of constitutive heterochromatin (H). Non-applicable data (-). Lateral bars correspond to chromosomes involved in complex rearrangements among different karyotypes.
