# Supplementary material for: Chromosomal phylogeny and comparative chromosome painting among Neacomys species (Rodentia, Sigmodontinae) from eastern Amazonia
Source: BMC Evol Biol. 2019 Oct 10;19:184. doi: 10.1186/s12862-019-1515-z (PMC6785907; doi:10.1186/s12862-019-1515-z)
Supplement: Supplementary file 5 — Additional file 5. Analysis and description of characters and characters states. [file 12862_2019_1515_MOESM5_ESM.docx]

**Analysis and description of characters and characters states**

Chromosomal morphology, number, and syntenic blocks based on hybridization with *Hylaeamys megacephalus* (HME) whole chromosome probes [10].

1. HME 1 chromosome. 0: with segments 1a, 1b and 1c together [1(a+b+c)]. 1: with segments 1a and 1(b+c) separated [1a+ 1(b+c)]. 2: with segments 1a, 1b and 1c separated (1a+1b+1c).
2. HME 2 chromosome. 0: with segments 2a, 2b and 2c together [2(a+b+c)]. 1: with segments 2a and 2(b+c) separated [2a + 2(b+c)]. 2: with segments 2a, 2b and 2c separated (2a + 2b + 2c).
3. HME 3 chromosome. 0: with segments 3a and 3b together [3(a+b)]. 1: with segments 3a and 3b separated (3a+3b).
4. HME 4 chromosome. 0: with segments 4a, 4b and 4c together [4(a+b+c)]. 1: with segments 4(a+c) and 4b separated [4(a+c) + 4b]. 2: with segments 4a, 4b and 4c separated (4a + 4b + 4c).
5. HME 5 chromosome. 0: with segments 5a, 5b, 5c and 5d together [5(a+b+c+d)]. 1: with segments 5(a+b) and 5(c+d) separated [5(a+b) + 5(c+d)]. 2: with segments 5(a+b), 5c and 5d separated [5(a+b) +5c +5d)]. 3: with segments 5a, 5b, 5c and 5d separated (5a+5b+5c+5d).
6. HME 6 chromosome. 0: with segments 6a, 6b and 6c together [6(a+b+c)]. 1: with segments 6a and 6(b+c) separated [6a+6(b+c)]. 2: with segments 6a, 6b and 6c separated (6a+6b+6c).
7. HME 7 chromosome. 0: with segments 7a and 7b together [7(a+b)]. 1: with segments 7a and 7b separated (7a+7b).
8. HME 8 chromosome. 0: with segments 8a and 8b together [8(a+b)]. 1: with segments 8a and 8b separated (8a+8b).
9. HME 11 chromosome. 0: with segments 11a and 11b together [11(a+b)]. 1: with segments 11a and 11b separated (11a+11b).
10. HME 14 chromosome. 0: with segments 14a and 14b together [14(a+b)]. 1: with segments 14a and 14b separated (14a+14b).
11. HME 18 chromosome. 0: with segments 18a, 18b and 18c together [18(a+b+c)]. 1: with segments 18a and 18(b+c) separated [18a+18(b+c)]. 2: with segments 18a, 18b and 18c separated (18a+18b+18c).
12. HME 19 chromosome. 0: with segments 19a and 19b together [19(a+b)]. 1: with segments 19a and 19b separated (19a+19b). 2: with segments 19a, 19b1 and 19b2 separated (19a+19b1+19b2).
13. HME 22 chromosome. 0: with segments 22a and 22b together [22(a+b)]. 1: with segments 22a and 22b separated (22a+22b).
14. HME 23 chromosome. 0: with segments 23a and 23b together [23(a+b)]. 1: with segments 23a and 23b separated (23a+23b).
15. HME 24 chromosome. 0: Metacentric. 1: Acrocentric. -: non-applicable character.
16. HME 25 chromosome (I). 0: with segments 25a and 25b together [25(a+b)]. 1: with segments 25a and 25b separated (25a+25b).
17. HME 25 chromosome (II). 0: Metacentric. 1: Acrocentric. -: non-applicable character.
18. HME 26 chromosome. 0: Metacentric. 1: Acrocentric. -: non-applicable character.
19. Chromosomal association HME 1/12. 0: Absence. 1: Presence.
20. Chromosomal association HME 1/20/[13,22]. 0: Absence. 1: Presence.
21. Chromosomal association HME 1/12/2/18/4/[16,17]/11. 0: Absence. 1: Presence.
22. Chromosomal association HME 18/2. 0: Absence. 1: Presence.
23. Chromosomal association HME 2/23. 0: Absence. 1: Presence.
24. Chromosomal association HME 3/25. 0: Absence. 1: Presence.
25. Chromosomal association HME 3/25/18. 0: Absence. 1: Presence.
26. Chromosomal association HME 4/18. 0: Absence. 1: Presence.
27. Chromosomal association HME 4/11/[16,17]. 0: Absence. 1: Presence.
28. Chromosomal association HME 8/[13,22]/5/[16,17]. 0: Absence. 1: Presence.
29. Chromosomal association HME 5/[9,10]. 0: Absence. 1: Presence.
30. Chromosomal association HME 5/[13,22]. 0: Absence. 1: Presence.
31. Chromosomal association HME 5/18. 0: Absence. 1: Presence.
32. Chromosomal association HME 5/11. 0: Absence. 1: Presence.
33. Chromosomal association HME [9,10]/14/5. 0: Absence. 1: Presence.
34. Chromosomal association HME 6/21. 0: Absence. 1: Presence.
35. Chromosomal association HME 6a/21. 0: Absence. 1: Presence.
36. Chromosomal association HME 7/[9,10]. 0: 7/[9,10]. 1: 7b/[9,10]. 2 [9,10]/7b/[9,10].
37. Chromosomal association HME [9,10]/15. 0: Absence. 1: Presence.
38. Chromosomal association HME [13,22]/11. 0: Absence. 1: Presence.
39. Chromosomal association HME 11/[16,17]. 0: Absence. 1: Presence.
40. Chromosomal association HME 12/[16,17]. 0: Absence. 1: Presence.
41. Chromosomal association HME 20/[13,22]. 0: Absence. 1: Presence.
42. Chromosomal association HME 20/[13,22]/4. 0: Absence. 1: Presence.
43. Chromosomal association HME 14/19. 0: Absence. 1: Presence.
44. Chromosomal association HME 19a/14/19b. 0: Absence. 1: Presence.
45. Chromosomal association 19a/14/19b1+19b2. 0: Absence. 1: Presence.
46. Chromosomal association 19a/14. 0: Absence. 1: Presence.
47. Chromosomal association HME 19/14/23. 0: Absence. 1: Presence.
48. Chromosomal association HME 19/7. 0: Absence. 1: Presence.
49. Chromosomal association HME 23/19/11. 0: Absence. 1: Presence.
50. Chromosomal association HME 3/25/6/21. 0: Absence. 1: Presence.
51. Chromosomal association HME [13,22]/26. 0: Absence. 1: Presence.
52. Chromosomal association HME [13,22]/21. 0: Absence. 1: Presence.
53. Chromosomal association HME 26/11. 0: Absence. 1: Presence.
54. Chromosomal association HME 5/19/14/19/5. 0: Absence. 1: Presence.
55. Chromosomal association HME 18/25. 0: Absence. 1: Presence.
56. Chromosomal association HME 24/5/[13,22]/11. 0: Absence. 1: Presence.
